# Dementia of the eye: the role of amyloid beta in retinal degeneration

**DOI:** 10.1038/eye.2015.100

**Published:** 2015-06-19

**Authors:** J A Ratnayaka, L C Serpell, A J Lotery

**Affiliations:** 1 Clinical and Experimental Science, Faculty of Medicine, University of Southampton, Southampton, UK; 2 School of Life Sciences (Biochemistry, Dementia Research Group), University of Sussex, Brighton, UK

## Abstract

Age-related macular degeneration (AMD) is one of the most common causes of irreversible blindness affecting nearly 50 million individuals globally. The disease is characterised by progressive loss of central vision, which has significant implications for quality of life concerns in an increasingly ageing population. AMD pathology manifests in the macula, a specialised region of the retina, which is responsible for central vision and perception of fine details. The underlying pathology of this complex degenerative disease is incompletely understood but includes both genetic as well as epigenetic risk factors. The recent discovery that amyloid beta (A*β*), a highly toxic and aggregate-prone family of peptides, is elevated in the ageing retina and is associated with AMD has opened up new perspectives on the aetiology of this debilitating blinding disease. Multiple studies now link A*β* with key stages of AMD progression, which is both exciting and potentially insightful, as this identifies a well-established toxic agent that aggressively targets cells in degenerative brains. Here, we review the most recent findings supporting the hypothesis that A*β* may be a key factor in AMD pathology. We describe how multiple A*β* reservoirs, now reported in the ageing eye, may target the cellular physiology of the retina as well as associated layers, and propose a mechanistic pathway of A*β*-mediated degenerative change leading to AMD.

## Introduction

Age-related macular degeneration (AMD) is a complex ocular disorder affecting a critical region of the retina known as the macula, which is crucial for central vision and perception of fine detail. The disease is the primary cause of irreversible blindness in societies with demographics favouring increasing age. The aetiology of this degenerative disorder is poorly understood, but contains both genetic as well as environmental risk factors.^1, 2, 3^ Central to degenerative pathology is the loss of visual function, which is associated with atrophy of photoreceptors and the underlying retinal pigment epithelium (RPE) that forms the blood–retinal barrier.^4, 5^ Retinal ganglion cells (RGC) and the RPE monolayer were recently identified as a major source of amyloid beta (A*β*) synthesis and secretion in the posterior eye.^6^ A*β* is a remarkably penetrative and highly toxic protein that aggressively targets neurons and is a key feature of neurodegenerative disease.^7, 8^ In the eye, multiple A*β* reservoirs were discovered in the retinal environment, while elevated A*β* levels were found in the ageing retina and linked with key stages of AMD progression.^6^ These findings support the hypothesis that A*β* has a crucial though previously uncharacterised role in driving degenerative processes in the ageing macula.

Here, we bring together the most recent findings emerging from the literature investigating AMD, neurodegeneration, as well as A*β*-structural biology, which support our hypothesis, and offer insights into fundamental degenerative events that could impair the senescent retina. A better understanding of how A*β* might target the retinal function may help in designing novel therapies to treat AMD in the future.

## Age-related macular degeneration

AMD is the most common cause of irreversible blindness in ageing societies, globally affecting ~50 million individuals with the direct cost estimated at nearly US$ 255 billion.^9^ The disease affects ~3% of adults,^10^ and notably increases to a quarter of the population by the eighth decade of life.^11^ A key process in vision loss is the gradual impairment of the RPE monolayer, which maintains photoreceptors on its apical surface and basally preserves the blood–retinal barrier.^1^ Early AMD is often asymptomatic, but is typified by the presence of sub-RPE deposits known as drusen consisting of cellular debris and lipids (including extracellular matrix constituents and inflammatory components).^12, 13^ Formation of hard drusen, which typically occur in the peripheral retina has well-defined borders, and is regarded to be a normal part of the ageing process. In contrast, the formation of macular soft drusen that is characterised by larger size, a diffuse nature with poorly-defined borders that rarely occurs before the age of 55 years is the first clinical indicator of increased risk of disease susceptibility.^5^ Late AMD is characterised by loss of central vision due to significant RPE/photoreceptor atrophy, referred to as ‘dry AMD', and/or the breakthrough of invasive blood vessels through the blood–retinal barrier referred to as ‘wet AMD'. Currently, the more prevalent dry form of the disease is untreatable, while several clinical strategies are used to treat the less common but more aggressive wet AMD, with varying degrees of success.^4^

Although AMD has been characterised clinically, the underlying mechanisms, especially during early disease, remain incompletely understood. The lack of molecular characterisation between dry and wet AMD has therefore limited our understanding and definition of the disease to largely clinical observations and terminology. The recent discovery of A*β* in the ageing retina and its link with AMD presents an exciting opportunity to view AMD from a new perspective, and to better understand disease onset and progression in novel molecular terms.

## A*β*—prevalence, structure, and dynamic assembly

**Figure 1:**
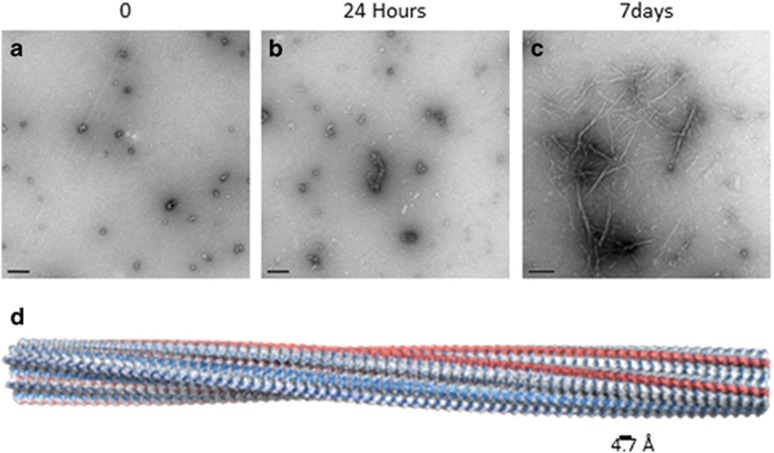
(a–c) Show negative stained transmission electron micrographs depicting the self-assembly of A*β*_1–42_ at pH 7.4, 50 *μ*M. (a) Shows small spherical oligomers that are visible immediately after preparation of A*β*_1-42_ peptide (Soura *et al*^50^). These assemble further by 24 h to form (b) elongated protofibrils, and finally (c) amyloid fibrils after 48 h incubation. d Shows a structural model of an amyloid fibril composed of cross-*β* structure and showing a slow twisted architecture. Generation of the model is described in Morris *et al.*^101^

The amyloid precursor protein (*APP*) gene located on chromosome 21q21 encodes a ubiquitously expressed integral type I membrane glycoprotein in several alternatively spliced forms, of which the most predominate isoforms include APP^751^, APP^770^, and APP^695^. APP transcripts and proteins are reported to be abundantly expressed in mouse, rat, as well as human RGC and RPE cells,^14, 15^ with APP^695^ being the principal isoform expressed in the brain.^16^ The function(s) of APP remain incompletely understood, with most studies suggesting signalling via several pathways in the brain.^17^ The proteolytic processing of APP occurs via two mutually exclusive routes referred to as the amyloidogenic and non-amyloidogenic (or constitutive) pathway.^18, 19^ Successive cleavage of APP in the amyloidogenic route by enzymes *β-* and *γ*-secretase produces the monomeric A*β* peptide with a molecular weight of ~4 kDa.^8, 20^ However, mutations in genes encoding APP and the enzyme presenilin, a component of the *γ*-secretase complex, promotes the generation of a longer isoform of A*β* that favours the amyloidogenic pathway, and is associated with several degenerative disease of the brain.^7, 21, 22^ A large body of work has focused on characterising the C-terminal cleavage of APP by *γ*-secretase that creates a heterogeneous mixture of A*β* peptides with different solubility, stability, and biological properties.^7, 8, 20^ Additional heterogeneity of A*β* peptides is generated by post-translational modifications mediated by aminopeptidases, glutaminyl-cyclase/isomerases, and by phosphorylation reactions resulting in a mixture of more than 20 A*β* species,^23, 24^ of which A*β*_37_, A*β*_38_, A*β*_40_, A*β*_42_, and A*β*_43_ have been reported in conditioned media of cells and in body fluids.^8^ The predominant forms of A*β* peptide are those with 40 and 42 residues, where A*β*_42_ generally forms fibrils more rapidly compared with the 40-residue species. This is considered to be due to the additional hydrophobic isoleucine and alanine residues at positions 41 and 42 in the peptide. Experimental substitution of these key amino acids with hydrophilic residues results in a decrease in assembly kinetics.^25^ Furthermore, hydrophobic regions of A*β*_42_ spanning residues 17–21 and 31–42 are considered to be important for fibril structure,^26^ while changes in the hydrophobicity in the sequence, for example, in the variant Phe20Glu alters A*β*_42_ toxicity, as well as the capacity to aggregate.^27^ Soluble, monomeric A*β* can be composed of *α*-helical and/or unordered structure, which then self-associates into low-molecular weight dimers, trimers, and oligomers with a conformational change to *β*-sheet. Further conformational changes result in the formation of higher ordered structures such as protofibrils and mature amyloid fibrils with a cross-*β* structural core^28^ (Figure 1). This sequence of events is described by the ‘nucleation-dependent polymerisation model', which proposes a 2-step process, where monomeric A*β* undergoes a slow thermodynamically unfavourable reaction to form oligomeric nuclei, followed by a rapid elongation/growth phase with the assembly of larger aggregates and fibril elongation. The formation of nuclei is the critical rate-limiting step, where further fibril formation can be significantly accelerated by the availability of preformed oligomers/nuclei.^29^ As the nucleus is the highest energy species in this reaction, its concentration should be very low during the aggregation time-course in contrast to monomers and fibrils. However, many studies show oligomer formation in the absence of detectable amyloid fibril formation early in the A*β* amyloidogenesis time course.^30, 31^ This has resulted in a mechanistic revision of the original model, and polymerisation of A*β* is proposed to occur via metastable intermediaries in a process referred to as ‘nucleated conformational conversion'.^32^

Studies utilising oligomeric A*β*, which impairs neurotransmission and causes neuronal death,^33^ as well as a close link between soluble A*β* oligomer levels and disease progression,^34, 35^ has resulted in a fundamental shift of interest from fibrillar A*β* to oligomeric A*β.*^8^ This shift in focus was highlighted in experiments utilising biomimetic unilamellar vesicles, which showed that as A*β* assembles from an oligomeric to fibrillar state, its ability to penetrate membranes also diminishes.^36^ Even relative to the monomeric form, oligomeric A*β* was found to preferentially interact with cellular membranes to become immobilised on the cell surface.^37^ The significant differences between oligomeric *vs* fibrillar A*β* has been proposed to be due to their different capacities to access intracellular compartments.^38^ This does not, however, automatically imply that fibrillar A*β* plaques are benign, as studies using AD mouse models show neurons in the vicinity of plaques to have reduced synaptic density, loss of synapses, as well as elevated resting Ca^2+^ levels.^39^ One hypothesis considers plaques as inert sinks; consisting of aberrantly folded proteins, lipids, and free metals, where a dynamic equilibrium between toxic A*β* oligomers and inert fibrils might exist, resulting in a local spillover of cytotoxic A*β* species in the vicinity.^8^ Consequently, age-related accumulation of such A*β* deposits may be viewed as potentially pathogenic reservoirs at critical locations in the retina and brain, which may contribute to chronic ‘local' A*β*-mediated toxicity, as well as associated inflammatory events characteristic of such degenerative tissues.

## Mechanisms of A*β* action in degenerative neurons

A*β* pathology in the ageing retina is not well understood. However, insights into A*β*-mediated mechanisms may be gleaned from studying degenerative brains where some mechanistic insights and pathways have been proposed with oligomeric A*β* as a key driver of pathogenicity. Commonly cited arguments against the role of A*β* in AD includes the lack of a correlation between A*β* plaques in AD brains and the extent of cognitive decline in Alzheimer's patients, as well as the observation that alterations to A*β* metabolism and appearance of amyloid plaques often occur many years before clinical symptoms.^40^ Nonetheless, degenerative neurons show a strong correlation with A*β*, a long-standing observation that is supported by a significant body of literature.^7, 8, 20, 23, 41, 42^ A*β* involvement in neurodegenerative and neurological spectrum disorders has been shown in Alzheimer's disease, Fragile X syndrome, Downs syndrome, Autism, Huntington's, and Parkinson's diseases.^7, 43^ AD brains, for example, are characterised by a marked neuronal loss and deposition of extracellular fibrils in neuritic plaques consisting of A*β* fibrils.^7, 20, 41^ The plethora of evidence is highly supportive, but does not prove the hypothesis that ill-defined soluble A*β* species are involved upstream in the pathogenic sequence of events that cause AD.^7, 8^ Some of these criticisms may be addressed by studying soluble A*β* oligomers, which demonstrate a much closer relationship with disease progression compared with amyloid plaques.^34, 35^ The relative importance of oligomeric *vs* fibrillar A*β* in degenerative retinas remains to be established.

Another feature of A*β* pathogenicity in AD is a critical shift in the relative A*β*_40_:A*β*_42_ ratios towards elevated A*β*_42_ that is correlated with increased disease susceptibility.^44^ However, A*β*-driven pathology is likely to be more complex and include both quantitative as well as qualitative changes to the spectrum of A*β* peptides.^8^ Age-related changes to A*β*_42_ as well as post-translational modifications, including pyroglutamate modifications, may well alter seeding of plaques, or drive independent cytotoxicity. Ultimately, the transient and complex nature of A*β* assemblies is an obstacle to elucidating the ‘toxic' A*β* species and/or conformation(s) that are detrimental to cellular physiology and function. Such issues are likely to arise when investigating A*β* mechanisms in the retina. This limited understanding of how A*β* assemblies cause pathogenicity also extends to mechanism(s) associated with A*β* cytotoxicity. The amphipathic nature of A*β* oligomers has been suggested to contribute to their ability to penetrate/coat/overlie the surface of cellular membranes, or potentially act as cell-penetrating peptides, and has been extensively reviewed elsewhere.^42^ As with most complex degenerative diseases, the impairment of cellular mechanisms is most likely to occur before appearance of senile plaques and onset of dementia. Indeed, a growing body of evidence supports the idea of early changes driven by toxic A*β* oligomers, including deterioration of long-term potentiation,^33^ microtubule abnormalities,^45^ as well as loss of synaptic function.^46^ The soluble A*β* fraction is primarily composed of A*β* monomers, dimer, trimers, and SDS-stable A*β* oligomers,^34, 44^ some of which has been reported in hippocampal CA1 region and the cortex of ageing human brains, even in the absence of senile plaques.^47^ The potency of these small A*β* assembles were highlighted in a study where introduction of soluble A*β* dimers and trimers into rodent brains resulted in cognitive impairment.^48^ Central to the idea of soluble toxic A*β* peptides as a driver of early pathogenicity is the initial entry of oligomeric A*β*, possibly via disruption of membrane integrity.^36, 49^ Our work has recently shown that oligomeric A*β* is rapidly internalised by neurons to accumulate in clathrin-positive endosomes,^50^ supporting evidence that clathrin-mediated endocytosis may be involved in A*β* internalisation,^51^ as well as findings showing inhibition of endosomal activity partially reduces A*β*-mediated toxicity.^52^ Other fundamental cellular mechanisms impaired by oligomeric A*β* in susceptible neurons are likely to include the impairment of axonal transport, mitochondrial dysfunction, and synaptic vesicle dynamics. Our ongoing studies to understand these key pathogenic changes will provide valuable insights into early A*β*-mediated activity in degenerative brains, as well as inform on potential pathways of damage in the retina.

## Evidence of A*β* in the ageing retina and AMD

### Constitutive A*β* generation in the normal retina

Both the retina and the central nervous system (CNS) share a common origin as both are derived from the developing neural tube. Both structures interface intimately with the adjacent vasculature via the blood–retinal and blood–brain barriers. Furthermore, with increasing age, both the retina and the brain develop extracellular deposits associated with degenerative pathology, referred to as drusen and senile plaques, respectively. It is therefore unsurprising that the many striking similarities between drusen and senile plaques include A*β*. Other shared components include the following; serum amyloid P component, apolipoprotein E, immunoglobulin, basement membrane matrices, proteoglycans, and metal ions (Fe^3+^, Cu^2+^, and Zn^2+^), acute-phase reactants, proteases/clearance-related elements, and several complement proteins, as well as other inflammatory mediators that are indicative of local inflammation typically associated with sub-retinal deposits.^6^ Such remarkable similarities between drusen and senile plaques, coincident with age and poor clinical prognosis, suggest that similar pathological mechanisms may drive degenerative changes in the retina as well as the brain.

Studies have now confirmed that RGC, the inner nuclear layer of the retina,^15, 53^ as well as the RPE^54^ expresses APP, and possesses the necessary cellular machinery to generate A*β*. Retinal and RPE cells expresses *β*-secretase, the four known subunits of *γ*-secretase, and the three major APP isoforms APP^770^, APP^751^, and APP^695^, as well as neprilysin.^14, 54, 55^ Furthermore, isolated RPE cells from wild-type C57BL/6 mice were shown to readily synthesise and secrete A*β*, which accumulated in conditioned media,^56^ while A*β* expression levels increased in rat RGC with age.^15^ This was not surprising, as abundant A*β*_40_ and A*β*_42_ peptides have been reported in both aqueous and vitreous humours. A*β* in ocular fluid is thought to originate primarily from the retina and RPE, from where it is secreted to the vitreous humour and subsequently transported to the anterior chamber.^55, 57^ This follows a similar pattern observed in the CNS, where A*β* is primarily synthesised in neurons but accumulate in cerebrospinal fluid (CSF).^58^ Not only does the retina and RPE constitutively express APP,^53, 55^ but RPE cells overlying, flanking, or displaced by drusen also show A*β* immunoreactivity in the cytoplasm.^54, 57^ Current measurements suggest that A*β* levels in the bovine vitreous and retina are considerably lower compared with CSF levels,^53, 55^ but this may reflect the dynamic behaviour of ‘local A*β* levels/species in the retina', as well as initial problems associated with A*β* quantification in these tissues. It is noteworthy that the retina is not only continuously exposed to A*β* species, but the very high concentrations of *α*-secretase cleaved soluble APP found in the vitreous fluids is comparable only to levels in CSF.^55^ Intriguingly, a recent study demonstrated that *α-* and *β*-secretase cleaved APP levels in conditioned media of wild-type neuronal cultures to be directly linked to extracellular A*β* concentrations, with a 1 : 1 relationship between *β*-cleavage of APP and release of A*β.*^59^ This highlights some of the difficulties in accurately quantifying A*β*, and given what is known about its pathogenicity in degenerative brains, provides further evidence that the retina is constitutively exposed to A*β* under normal/healthy conditions.

### The retinal A*β* burden increases with age

Growth of A*β* deposits with advancing age may be viewed as an alteration in the balance between increased A*β* synthesis *vs* a reduction in the ability to clear such aggregates. Either or both fates may be sufficient to elevate the A*β* burden in the ageing retina. For example, cultured RPE cells from geriatric C57BL/6 mice displayed elevated A*β* levels in conditioned media compared with RPE cells from younger controls. In contrast, mRNA levels of neprilysin (which clears A*β*) were significantly decreased, while *β*-secretase activity was elevated in senescent RPE cells, indicating the ability to clear A*β* also diminished with age.^56^ Analysis of C57BL/6 mice as young as 3 months by immunofluorescence and immunoblotting techniques revealed A*β* accumulations in the RPE-Bruch's membrane interface, as well as in retinal/choroidal blood vessels. With age, A*β* accumulations in the critical RPE-Bruch's region increased in subsequent months.^60^ Of note, this pattern of amyloid deposition was observed in a region where A*β* accumulation is thought to first occur in AMD; in close proximity to the inner collagenous layer of Bruch's membrane.^61^ Interestingly, A*β* staining in inner retinal vessels appeared discontinuous, while A*β* positivity in the choroidal vasculature were confined to sub-groups of vessels suggesting a degree of selectivity in A*β* deposition.^60^ Such points of vulnerability may be related to thinning of blood vessels and reduced flow rates, as observed in the retinal vasculature of early AD patients.^62^

**Figure 2:**
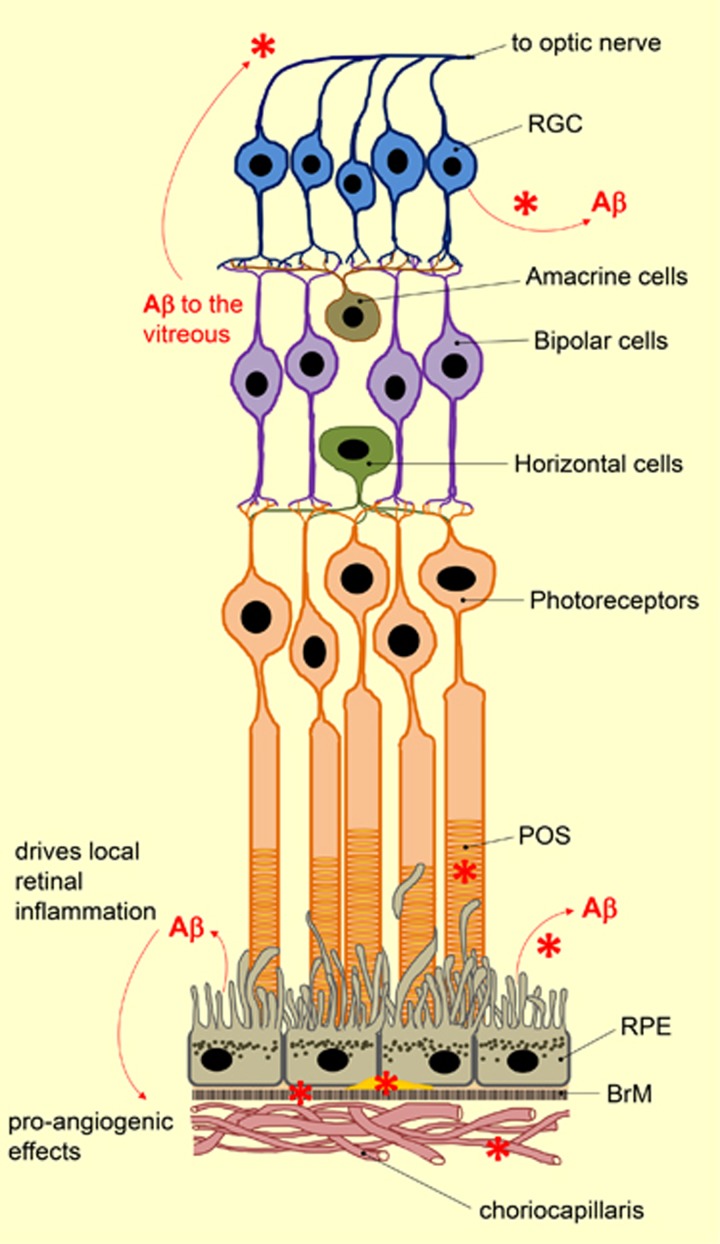
Schematic diagram showing multiple locations of A*β* synthesis, secretion, and aggregation in the ageing retina (red asterisk) that is reported in the literature to date. BrM, Bruch's Membrane; POS, photoreceptor outer segments; RGC, retinal ganglion cells; RPE, retinal pigment epithelium.

A*β* deposition with increasing age was not restricted to sub-RPE regions, but was unexpectedly discovered to accumulate in photoreceptor outer segments (POS) in older mice. Such deposits were identified as early as 3 months and by 12 months the outer segments were completely wrapped in A*β*-containing material, which appeared qualitatively different by 24 months.^60^ Although there is no direct evidence that such material is purely A*β*, the close association between A*β*-immunostaining patterns and scanning EM images argue that A*β* at least constitutes an element of such age-related deposits. Additionally, intravitreal injection of oligomeric A*β*_40_ into wild-type rats produced the highest immunostaining intensity levels in POS, supporting the idea of preferential A*β* accumulation in the apical proximity of RPE cells.^63^ Analysis of human post-mortem samples between ages of 31 and 90 years mirrored a similar pattern of increasing A*β* immunostaining in POS.^60^ This pattern of A*β* accumulation originating at the apical tip of POS and progressing along its length illustrate-specific A*β* aggregation,^60^ and as such, agree with other findings^14, 54, 55, 56^ showing retinal/RPE generated A*β* accumulating in posterior eye with advancing age (Figure 2).

### A*β* aggregation is involved in key stages of AMD

The evidence discussed thus far confirms that A*β* has a central role in AMD, a large part of which is derived from human post-mortem eyes,^57, 61, 64^ and provides a clinical snapshot of A*β* involvement in key stages of disease progression. Although such data are often based on high-quality static images, they nonetheless portray a tantalising picture of dynamic A*β* activity in the ageing retina. The presence of A*β* in the retina appears to be correlated with age as well as the extent of sub-retinal drusen loads. For example, EM analysis of 152 human donor eyes ranging from 9 to 91 years of age showed that A*β* assemblies were most prevalent in retinas with moderate-to-high drusen loads.^57^ Although links between sub-RPE A*β* deposition in the macula *vs* peripheral retina, or early *vs* late AMD, or indeed with specific disease phenotypes were not examined, these findings suggest that A*β* might be associated with more advanced stages of AMD. A smaller study consisting of nine AMD retinas and an equivalent number of control retinas found that drusen containing A*β* were present only in patients with AMD. Elevated A*β* reactivity was detected in four of nine AMD retinas, with a few A*β*-positive drusen in two early AMD retinas and numerous A*β*-positive drusen in two retinal samples with geographic atrophy.^65^ Although this study lacked sufficient sample numbers to arrive at any firm conclusions linking A*β*-positive drusen with AMD, it nonetheless suggests that A*β* pathogenicity is involved in distinct stages of AMD. Confocal immunofluorescence and ultrastructural analysis of post-mortem retinas revealed that A*β* is localised in sub-structural vesicular components within drusen referred to as ‘amyloid vesicles'.^54, 57, 61, 64^ These structures ranged from 2 to 10 *μ*m in diameter and were readily detected in both macular and peripheral drusen from donors with/without clinical AMD.^54^ Such amyloid-containing structures within drusen have been reported by several groups using a variety of different A*β* antibodies and appear to vary between 0.25–10 *μ*m,^57^ 10–15 *μ*m,^61^ and 10–20 *μ*m in diameter.^64^ In addition, the relative shapes of such amyloid structures within drusen also varied; from spheres to elongated forms,^57, 61^ and to vesicles that appear to be in the process of budding or fusing.^54^ Although all studies were in agreement that each drusen may contain multiple amyloid structures, descriptions of amyloid cores and vesicles were largely defined by the choice of A*β* antibodies used in the respective studies.^54, 57, 61, 64^ Some drusen were described as densely packed with amyloid vesicles accounting for a significant proportion of their total volume, while others contained only a single large vesicle that occupied a substantial portion of the drusen mass.^54^ For example, Anderson *et al*^57^ reported a single drusen to contain >100 spheres of various sizes. The presence of multiple amyloid cores in larger drusen suggested that these drusen may have formed from a coalescence of smaller drusen,^61^ indicating the evolving complexity of A*β* containing drusen over long periods of time.

**Figure 3:**
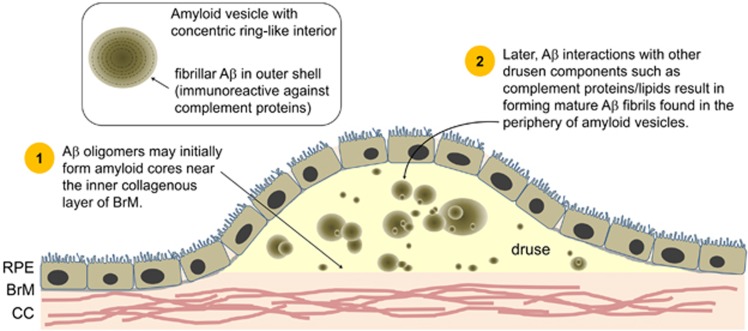
A*β* assemblies are prevalent in individuals with moderate-to-high levels of drusen. A*β* is organised into spherical structures termed ‘amyloid vesicles', which can occupy a large portion of drusen volume, and form potential sites of complement activation. A druse may be densely packed with several amyloid vesicles or contain only a single large vesicle. The presence of distinct A*β* structures may reflect the evolving nature of drusen with disease progression. For example, the presence of multiple oligomer cores in a large drusen may be due to smaller drusen coalescing over time. A*β* in drusen correlates with degenerating photoreceptors and RPE. CC, choriocapillaris; BrM, Bruch's membrane; RPE, retinal pigment epithelium.

Further analysis of amyloid vesicles revealed a highly organised interior consisting of concentric ring-like layers with varying electron densities and bound by an electron dense shell of ~100 nm thick.^57^ A similar description have also been made with the vesicle interior described as consisting of flocculent material and/or concentric ring-like elements bound by an outer shell or vesicle rim.^54^ A*β* immunoreactivity was detected throughout all the layers, signifying the apparent central role of A*β* in amyloid vesicles within drusen.^57^ Despite the limited scope of data offered by human post-mortem eyes along a single plane, as well as a singular point in time, they nonetheless show that A*β* antibodies specific for different conformations localise to different parts of amyloid structures.^61, 64^ For example, the A11 and M204 antibodies that specifically recognise the toxic oligomeric A*β* forms, but not A*β* monomers or fibrils were typically found to localise centrally within drusen in close proximity to the inner collagenous layer of Bruch's membrane.^61, 64^ Hence, the authors believe that such oligomeric cores are different to the substructures described by Anderson *et al*,^57, 61^ but conclude that they nonetheless form the majority of A*β* structures observed in drusen.^64^ Additionally, a wide spectrum of antibodies such as OC, 6E10, WO1, WO2, and 4G8, which specifically bind to A*β* protofibrils and mature fibrils showed a propensity to accumulate towards the outer periphery and shell of amyloid structures within drusen.^54, 57, 61, 64^ Hence, despite the lack of a comprehensive study that systematically investigates the full spectrum of A*β* conformations in retinal substructures of human *mort-mortem* retinas, the collective findings thus far agree that drusen contain an abundant variety of A*β* forms and structures (Figure 3).

### A*β* deposits in the retina triggers a pro-inflammatory and pro-angiogenic microenvironment

The experimental exposure of cells in the retina, RPE, and choroid to A*β* can induce fundamental changes associated with local retinal inflammation. This evidence is derived from a variety of experimental culture systems, as well as from animal models including Zebra fish, rabbits, rodents, and human post-mortem eyes. A systematic review of these findings reveals a progressive pattern of A*β*-mediated inflammatory and pro-angiogenic effects in the ageing retina, in which we are able to discern between early A*β*-driven changes as well as late-stage AMD pathology associated with A*β*. Such changes are likely to be triggered, and chronically sustained, by a toxic cocktail of A*β* peptides that is readily supplied by multiple A*β* reservoirs surrounding the ageing retina, which includes the immediate environment around the RPE,^56^ in vitreous fluid,^55, 57^ the coating of the outer segments of photoreceptors,^60^ and in sub-retinal drusen.^54, 57^ Furthermore, these early events are likely to occur well in advance of clinical AMD and include alterations in the expression profiles of key inflammatory genes. For example, human foetal RPE cultures treated with nanomolar concentrations of oligomeric A*β*_40_ for as little as 24 h resulted in a significant upregulation of pro-inflammatory cytokines IL-1*β* and IL-8.^66^ Another study also demonstrated IL-8 as well as MMP-9 overexpression following oligomeric A*β*_40_ treatment, coincident with RPE senescence and compromised barrier properties.^67^ The role of IL-1*β* in generating reactive oxygen species (ROS) as well as IL-8 in RPE cells has been previously documented, while IL-8 itself is a potent inducer of chemotaxis, correlated with amplification of inflammatory responses and neovascularisation.^68, 69^ Exposure of the D407 RPE cell line to oligomeric A*β*_40_ resulted in elevated IL-33, which can accelerate the production of Th2-associated cytokines and promote tissue inflammation.^70^ Elevation of oxidative stress responses in cultured ARPE-19 cells were also observed within hours of treatment using nanomolar to micromolar levels of the more toxic oligomeric A*β*_42._^71^ These pro-inflammatory activities driven by A*β* are not limited to cultures but are also replicated in animal models. For example, the use of wild-type rats to investigate acute effects of A*β*_40_ following intravitreal injections revealed elevated levels of pro-inflammatory IL-1*β*, IL-6, IL-8, and TNF-*α* in the RPE/choroid and neuroretina. In addition, elevation of caspase-1 and NLRP3 indicated activation of the retinal/RPE inflammasome,^63^ which has been implicated in AMD susceptibility.^72^ The varying fates of retinal/RPE cells following acute application of A*β in vivo* may reflect a mixture of varied A*β* cytotoxicity as well as A*β*-dosages, length of treatment as well as sites of injection. Hence, intravitreal A*β*_40_ injections failed to show significant retinal/RPE cell death,^63^ which is in stark contrast to RPE hypopigmentation, disorganised photoreceptors/RPE, and halving of photoreceptor numbers soon after sub-retinal injections of oligomeric A*β*_42_ into wild-type mice.^71^ Similarly, RGC cultures acutely treated with A*β*_25–35_ or A*β*_1–42_ induced apoptosis at micromolar concentrations, while treatment with A*β*_1–40_ proved less toxic.^73^ The pattern of RGC apoptosis was also observed in a mouse model of glaucoma-associated A*β* co-localisation,^74^ highlighting the potential involvement of A*β* in multiple degenerative conditions in the eye. Taken together, these findings demonstrate that key changes in gene expression of retinal and RPE cells mediated by A*β* are replicated *in vivo* to promote a pro-inflammatory milieu in early AMD pathogenesis.

The complement system consist of regulatory molecules in systemic circulation, which constitute the classical, alternative as well as the lectin pathways, and has an important role in AMD susceptibility and risk of disease progression.^75^ These distinct mechanism of the complement system converge on a common terminal pathway culminating in the formation of the membrane attack complex (MAC), opsonization, and lysis of target cells as well as the recruitment and/or activation of inflammatory cells.^75, 76^ The ability of A*β* peptides to induce chronic inflammation in degenerative brains via direct and independent activation of the complement pathway has been well established.^77, 78^ Evidence now supports the possibility that A*β*, specifically around drusen, can mediate early inflammatory events in degenerative retinas in a similar manner. An insight into such mechanisms is provided by studies of human post-mortem eyes, showing RPE-synthesised factor H (HF1), a major regulator of the alternative complement pathway, co-localising with its ligand C3b/iC3b in amyloid-containing vesicles within drusen. HF1 and MAC accumulated along the surface of amyloid vesicles in the RPE-choroidal interface and were prevalent in the macular regions from donors with prior histories of AMD.^79^ This association was also shown in another study of human post-mortem retinas, which revealed iC3b, the activated product of complement C3 in close proximity and co-localised with A*β* deposits in amyloid vesicles.^54^ A*β* deposits in drusen may form a nucleus around which chronic ‘wound-like' events may occur (Figure 3), a model which builds on an elegant hypothesis that chronic local inflammatory and immune-mediated events at the level of the RPE-Bruch's membrane have a critical role in drusen biogenesis, and in the pathobiology of AMD.^13, 54, 76, 79^

The RPE has a central role in maintaining the blood–retinal barrier, an important function in sustaining the immune-privileged status and homoeostasis of the retinal environment.^1, 5^ A*β*-mediated pathogenicity in early AMD may also target the structural integrity of RPE barrier properties. For example, acute treatment of ARPE-19 monolayers with 0.1–10 *μ*M oligomeric A*β*_42_ resulted in the disruption of RPE junctional complexes and actin cytoskeleton, formation of actin stress fibres, impairment of trans-epithelial permeability as well as loss of cell attachment.^71^ Similar studies in human foetal RPE cultures found that exposure to A*β*_42_ elevated MMP-9 secretion and shifted cells into a senescent state.^67^ These findings show a systematic breakdown of ZO-1 and occludin junctional complexes within the RPE that is mediated by MMP-9, and suggest an early mechanism by which chemokine gradients can be established across barriers for subsequent migration of inflammatory cells. Such MMP-driven mechanistic changes have been previously documented in retinas of patients with AMD.^80^ A*β*-driven structural changes in the RPE monolayer were also observed *in vivo*, following sub-retinal injections of oligomeric A*β*_42_, and consisted of disorganised actin filaments and junctional complexes in the absence of apoptosis. Further changes observed include RPE hypopigmentation, damage to photoreceptors including loss of outer segments, and shorter inner segments.^71^ The complexities of A*β*-mediated activities also include the capacity to generate elevated ROS, a well-documented process in degenerative brains.^81^ The retina, which is normally subject to constantly high photoxidative stresses,^82^ may be particularly prone to A*β*-induced ROS-induced damage, in a process compounded by increasing lipofuscin accumulation within RPE cells with age. Furthermore, A*β* has been shown to induce cellular senescence and impair mitochondrial activity in RPE cells.^56, 67, 71^ The substantial impact of accumulated mitochondrial damage in post-mitotic RPE cells has been well documented in AMD susceptibility.^83^

Late-stage A*β*-driven mechanisms in AMD may be considered cumulative, and most likely to occur after decades of chronic A*β* exposure in the ageing retina. Although we do not yet fully understand the extent of these mechanisms, a numbers of studies provide a tantalising insight. For instance, exposure of human RPE cultures to 1–25 *μ*M A*β*_40_ for as little as 24 h resulted in a significant increase of pro-angiogenic VEGF (vascular endothelial growth factor) expression and a concomitant decrease in anti-angiogenic PEDF (pigment epithelium derived factor).^14^ The role of VEGF in increasing vascular permeability and triggering endothelial cell proliferation has been well established.^1, 5, 14^ The RPE monolayer appears to be the only source of VEGF in the retinal environment, and secretes several VEGF isoforms through its basolateral surface towards the choriocapillaris.^82^ VEGF has a key role in the development of CNV, with anti-VEGF treatment currently forming the mainstay of treatment for wet AMD.^1^ The ability of A*β* peptides to elevate VEGF levels in the vicinity of the blood–retinal barrier via direct mechanisms^14^ and possibly via indirect inflammatory triggers^54, 63, 70, 76^ may help explain the as yet incompletely understood pathology underlying VEGF elevation preceding neovascularisation. Furthermore, conditioned media from RPE cells exposed to A*β*_40_ triggered tube formation in human umbilical vein endothelial cells, suggesting that A*β* also has the capacity to directly influence CNV.^14^ Such direct pro-angiogenic mechanisms were further confirmed when injection of A*β*_42_ into Zebra fish eyes resulted in a significant increase of retinal capillary bed densities.^84^ Collectively, these findings demonstrate that A*β* peptides have a central role in driving early as well as late-stage degenerative mechanisms in the ageing retina.

### AMD risk factors promotes A*β* aggregation in the ageing retina

The heterogeneous nature AMD aetiology suggests that the most likely scenario involves a convergence of multiple risk factors to trigger disease pathology in the ageing retina.^1, 5, 85^ One intriguing idea is that other well-characterised AMD/AD risk factors may have a supportive role in exacerbating A*β* pathology in the eye. These may include a combination of genetic as well as epigenetic risk factors such as diet. Studies to elucidate the molecular basis underlying these changes illustrate striking parallels of A*β*-mediated damage common to both retina and brain. For example, A*β*-mediated disruption of the blood–retinal barrier^71, 80^ is mirrored in the blood–brain barriers of Tg2576 mice. Specifically, overexpression of A*β*_42_ in these mice resulted in significant disruption of tight junctions in the cerebral vasculature, long before consolidation of amyloid plaques.^86^ However, it must be noted that genetic risk factors driving pathology in one compartment may not necessary act in an identical manner in another location. ApoE, which encodes a glycoprotein responsible for cholesterol transport is highly expressed in the retina and is likely to have an important role in maintaining normal retinal function. The frequency of ApoE alleles (*ɛ*2, *ɛ*3, and *ɛ*4) displays a diverging story in AMD and AD. In AD, for example, the *ɛ*4 allele confers a dose-dependent elevated risk with a decrease in the mean age of AD onset. In contrast, the *ɛ*2 allele has a beneficial effect on disease-free time, and appears to impart a measure of protection.^7, 8^ In contrast, our studies using pooled analysis of a large data set of both published and previously unreported studies shown that *ɛ*4 protects against late AMD. We also reported an increased risk for late AMD in individuals homozygous for *ɛ*2.^87^ Such contrasting effects may reflect significant variations in the local structure and physiologies in the senescent brain and retina, respectively. For instance, the positively charged *ɛ*4 haplotype has been proposed to improve permeability of Bruch's membrane, which could facilitate lipid transport and reduce sub-retinal debris accumulation associated with drusen formation.^12^ Reduced transport of lipoprotein across Bruch's membrane is a consequence of ageing, and has been proposed to promote drusen deposition and impairment of the RPE.^88^ Furthermore, the *ɛ*4 isoform has also been implicated in the transport of macular pigments lutein and zeaxanthin, the reduced dietary intake of which is associated with increased risk of AMD.^89^ An important factor regulating ApoE effects is their interaction with A*β*. In the past, distinct binding properties of different ApoE isoforms to A*β* has been suggested to underlie the discrepancies associated with each genotype,^90^ while more recently, ApoE isoforms were shown to affect A*β* clearance^91^ and/or oligomerisation,^92^ which could lead to diverging outcomes in different tissues.

Cholesterol forms a vital component of the eukaryotic cell regulating membrane fluidity, permeability, and electrical properties. Evidence supports the possibility that the cholesterol content of specific anatomically defined locations in the brain may leave some regions particularly vulnerable in old age. For example, a significant reduction of cholesterol levels in the temporal gyrus of AD brains *vs* non-demented brains could result in increased A*β* permeation. Furthermore, the decreased cholesterol/phospholipid ratio in AD brains may affect APP cleavage and elevate A*β* generation, as well as facilitate increased A*β* permeability.^42^ Studies using mouse models have previously shown that a cholesterol enriched diet dramatically exacerbated A*β* pathology, whereas cholesterol-lowering drugs decreased the A*β* burden as well as AD pathology.^93^ Although the precise nature of interaction(s) between cholesterol and A*β* is not fully understood,^93^ there is evidence to suggest that cholesterol has the capacity to modulate A*β* generation and its clearance.^94^ Similar effects were observed when RPE cells obtained from C57BL/6 mouse were treated with cholesterol resulted in a significantly increased A*β* production, while activities of A*β*-degrading enzyme neprilysin and anti-amyloidogenic *α*-secretase showed a concomitant decrease. Furthermore, senescent C57BL/6 wild-type mice fed with a cholesterol enriched diet developed sub-RPE deposits containing A*β.*^95^ This pattern of cholesterol-driven A*β* pathology was also observed when New Zealand white rabbits were switched to a cholesterol enriched diet. A*β* deposition was detected in POS, in the outer and inner nuclear layers, as well as in RGC. This was accompanied by increased A*β*_40_ and A*β*_42_ levels in retinal samples as quantified by ELISA measurements. Further changes include drusen-like debris, increased generation of ROS and apoptotic retinal cells.^96^ The high dietary intake of cholesterol and saturated fat has been regarded as AMD risk factors for a long time. This is coupled with the observation that cholesterol forms a major component of drusen, the ageing Bruch's membrane and sub-retinal lesions.^88^ As most drusen components are thought to be primarily derived from the retinal environment,^12, 88^ implications for the interplay between A*β* and cholesterol is an intriguing possibility.

## Animal models of AMD and anti-A*β* antibody therapy

As an age-related degenerative disease with a complex aetiology, the full spectrum of AMD pathology has been challenging to reproduce in animal models. This, however, has not prevented the development of numerous rodent, rabbit, porcine, and non-human primate animal models. The widely utilised mouse/rat models have the benefit of lower costs, ease of maintenance and the capacity to develop disease symptoms in a relatively short time period, but suffers from defects including the most glaring of which is the lack of a macula. Critically, no single model has been successful in reproducing the full disease spectrum of AMD, although convincing models exist that reproduce limited features of both geographic and exudative forms of the disease. In contrast, the use of non-human primates offers the opportunity to study AMD in a system bearing closer resemblance and physiology to humans, but carries a number of disadvantages including considerable ethical implications, difficulties in genetic manipulation, as well as longer time scales prior to disease onset.

Recent findings that the A*β* burden considerably increases in the senescent retina, and that A*β* may have a central role in drusen formation, has revealed yet another feature that needs to be reproduced *in vivo*. As with other key milestones of disease progression, the development of an A*β* phenotype along with drusen formation, RPE/photoreceptor abnormalities, CNV, and progressive visual impairment may be critical in ultimately developing a *bona fide* model of AMD. Unfortunately, only a limited number of rodent models are currently in existence that reproduce retinal A*β* abnormalities. One of the first animal models to place A*β* in the centre stage of retinal pathology was a neprilysin-deficient senescent mouse model that developed A*β*-containing drusen, changes in the outer retina, as well as RPE abnormalities.^14^ This model also exhibited elevated VEGF expression and diminished PEDF levels suggesting a shift into a pro-angiogenic phenotype, but surprisingly failed to develop CNV even at the advanced age of 27 months. The authors concluded that the lack of progression to late-stage AMD was likely due to the insufficient Bruch's membrane abnormalities, differences in the role of complement activation or that the animals were insufficiently aged to mimic the senescent human retina. Nonetheless, this mouse model represents a useful tool to study A*β*-driven pathology in early disease.^14^

The senescent human APOE4 knock-in mouse represents another intriguing model, which upon switching to a high-fat cholesterol-rich (HFC) diet develops drusen, thickened Bruch's membrane, abnormal RPE adjacent to degenerative photoreceptors, and in extreme cases CNV.^97^ Of note, A*β* deposits were associated with sub-RPE drusen and with neovascular vessels, with mice developing visual impairment as measured by electroretinogram. Although this model represents an excellent tool to investigate A*β*-mediated pathology in the mouse retina in its own right, the most pertinent finding was that a dose-dependent systemic administration of antibodies targeting the C-terminal of both A*β*_40_ and A*β*_42_ in APOE4-HFC mice resulted in a significant protective effect.^98^ Hence, immunised age-matched animals showed reduced A*β* and activated complement components in sub-RPE deposits, improved structural integrity of the RPE monolayer as well as visual protection. This follows the pattern of reduced amyloid plaques and improved cognitive function in mouse models of AD treated with anti-A*β* antibodies.^8, 20^ The importance of A*β* in driving retinal degenerative events that manifest as different pathologies was also demonstrated in a mouse models of glaucoma, where A*β*-neutralising antibody treatment produced >80% reduction in RGC apoptosis.^74^ Collectively, such evidence firmly places A*β* at the centre stage of degenerative events in the ageing retina.

## A*β* and AMD—lessons from neurodegeneration and a way forward

Developed societies are confronted with new challenges as the numbers of older individuals gradually begin to outstrip the younger age groups. The impact of age-related illnesses such as dementia, AMD, cardiovascular disease, and osteoporosis are felt at many levels; from individuals to families and societies, and have a major role in setting government health policy. In the UK, AMD affects a significant proportion of the elderly, as well as adults that are registered legally blind. For patients with nvAMD, anti-VEGF treatment offers scope for burdensome disease management through repeated hospital visits consisting of monthly intravitreal injections. However, not all respond to this therapy, while at present, the majority of AMD patients have no effective treatment. The complex disease aetiology of AMD poses major challenges to devising effective solutions. Recent advances in understanding the genetic architecture of AMD has yet to translate to meaningful benefits for patients. An incomplete understanding of the biological processors underpinning disease mechanisms largely accounts for this critical knowledge gap. Degenerative processes in the ageing retina and brain show striking similarities, and offers scope for identifying novel targets as well as pathogenic mechanisms. A*β*, a highly toxic and aggregate-prone peptide capable of eliciting local inflammation and involved in key stages of AMD can be considered such a candidate.

Here, we discussed the hypothesis and exciting new findings that show A*β* has the capacity to play a key role in AMD, the study of which may offer a better understanding of early disease mechanisms, as well as molecular pathways sustaining chronic retinal degeneration. Examples of shared pathology in AD patients include reduced thickness of the nerve fibre layers,^99^ abnormal retinal blood circulation,^62^ as well as reduced choroidal thickness,^100^ locations where degeneration also occurs in glaucoma and AMD.^1, 5, 74^ Similarities are also found between AD senile plaques and AMD drusen,^6^ as well as the pattern of selective tissue damage, which argues for shared molecular mechanisms in at least some stages of these diseases. Studies of A*β* and associated pathology in the retina have the potential to offer new insights into AMD, and approach this debilitating blinding disease from a new perspective. Such investigations are already underway in our laboratory.

## References

[bib1] Khandhadia S Cherry J Lotery AJ Age-related macular degeneration Adv Exp Med Biol 2012 724 15 36 22411231 10.1007/978-1-4614-0653-2_2

[bib2] Cipriani V Leung HT Plagnol V Bunce C Khan JC Shahid H Genome-wide association study of age-related macular degeneration identifies associated variants in the TNXB-FKBPL-NOTCH4 region of chromosome 6p21.3 Hum Mol Genet 2012 21 (18 4138 4150 22694956 10.1093/hmg/dds225 PMC3428154

[bib3] Fritsche LG Chen W Schu M Yaspan BL Yu Y Thorleifsson G Seven new loci associated with age-related macular degeneration Nat Genet 2013 45 (4 433 2 23455636 10.1038/ng.2578 PMC3739472

[bib4] Lotery A Progress in understanding and treating age-related macular degeneration Eye (Lond) 2008 22 (6 739 741 18548082 10.1038/sj.eye.6703035

[bib5] Lotery A Trump D Progress in defining the molecular biology of age related macular degeneration Hum Genet 2007 122 (3-4 219 236 17659362 10.1007/s00439-007-0406-3

[bib6] Ohno-Matsui K Parallel findings in age-related macular degeneration and Alzheimer's disease Prog Retin Eye Res 2011 30 (4 217 238 21440663 10.1016/j.preteyeres.2011.02.004

[bib7] Hardy J Selkoe DJ The amyloid hypothesis of Alzheimer's disease: progress and problems on the road to therapeutics Science 2002 297 (5580 353 356 12130773 10.1126/science.1072994

[bib8] Benilova I Karran E De SB The toxic Abeta oligomer and Alzheimer's disease: an emperor in need of clothes Nat Neurosci 2012 15 (3 349 357 22286176 10.1038/nn.3028

[bib9] Gordois A Cutler H Pezzullo L Gordon K Cruess A Winyard S An estimation of the worldwide economic and health burden of visual impairment Glob Public Health 2012 7 (5 465 481 22136197 10.1080/17441692.2011.634815

[bib10] Klein R Cruickshanks KJ Nash SD Krantz EM Nieto FJ Huang GH The prevalence of age-related macular degeneration and associated risk factors Arch Ophthalmol 2010 128 (6 750 758 20547953 10.1001/archophthalmol.2010.92 PMC2896217

[bib11] Friedman DS O'Colmain BJ Munoz B Tomany SC McCarty C de Jong PT Prevalence of age-related macular degeneration in the United States Arch Ophthalmol 2004 122 (4 564 572 15078675 10.1001/archopht.122.4.564

[bib12] Crabb JW Miyagi M Gu X Shadrach K West KA Sakaguchi H Drusen proteome analysis: an approach to the etiology of age-related macular degeneration Proc Natl Acad Sci USA 2002 99 (23 14682 14687 12391305 10.1073/pnas.222551899 PMC137479

[bib13] Anderson DH Mullins RF Hageman GS Johnson LV A role for local inflammation in the formation of drusen in the aging eye Am J Ophthalmol 2002 134 (3 411 431 12208254 10.1016/s0002-9394(02)01624-0

[bib14] Yoshida T Ohno-Matsui K Ichinose S Sato T Iwata N Saido TC The potential role of amyloid beta in the pathogenesis of age-related macular degeneration J Clin Invest 2005 115 (10 2793 2800 16167083 10.1172/JCI24635 PMC1201663

[bib15] Wang J Zhu C Xu Y Liu B Wang M Wu K Development and expression of amyloid-beta peptide 42 in retinal ganglion cells in rats Anat Rec (Hoboken) 2011 294 (8 1401 1405 21717587 10.1002/ar.21438

[bib16] Yoshikai S Sasaki H Doh-ura K Furuya H Sakaki Y Genomic organization of the human amyloid beta-protein precursor gene Gene 1990 87 (2 257 263 2110105 10.1016/0378-1119(90)90310-n

[bib17] Shariati SA De SB Redundancy and divergence in the amyloid precursor protein family FEBS Lett 2013 587 (13 2036 2045 23707420 10.1016/j.febslet.2013.05.026

[bib18] Shoji M Golde TE Ghiso J Cheung TT Estus S Shaffer LM Production of the Alzheimer amyloid beta protein by normal proteolytic processing Science 1992 258 (5079 126 129 1439760 10.1126/science.1439760

[bib19] Haass C Schlossmacher MG Hung AY Vigo-Pelfrey C Mellon A Ostaszewski BL Amyloid beta-peptide is produced by cultured cells during normal metabolism Nature 1992 359 (6393 322 325 1383826 10.1038/359322a0

[bib20] Haass C Selkoe DJ Soluble protein oligomers in neurodegeneration: lessons from the Alzheimer's amyloid beta-peptide Nat Rev Mol Cell Biol 2007 8 (2 101 112 17245412 10.1038/nrm2101

[bib21] Suzuki N Cheung TT Cai XD Odaka A Otvos L Jr Eckman C An increased percentage of long amyloid beta protein secreted by familial amyloid beta protein precursor (beta APP717) mutants Science 1994 264 (5163 1336 1340 8191290 10.1126/science.8191290

[bib22] Citron M Oltersdorf T Haass C McConlogue L Hung AY Seubert P Mutation of the beta-amyloid precursor protein in familial Alzheimer's disease increases beta-protein production Nature 1992 360 (6405 672 674 1465129 10.1038/360672a0

[bib23] De SB Proteases and proteolysis in Alzheimer disease: a multifactorial view on the disease process Physiol Rev 2010 90 (2 465 494 20393191 10.1152/physrev.00023.2009

[bib24] Kumar S Rezaei-Ghaleh N Terwel D Thal DR Richard M Hoch M Extracellular phosphorylation of the amyloid beta-peptide promotes formation of toxic aggregates during the pathogenesis of Alzheimer's disease EMBO J 2011 30 (11 2255 2265 21527912 10.1038/emboj.2011.138 PMC3117653

[bib25] Kim W Hecht MH Sequence determinants of enhanced amyloidogenicity of Alzheimer A{beta}42 peptide relative to A{beta}40 J Biol Chem 2005 280 (41 35069 35076 16079141 10.1074/jbc.M505763200

[bib26] Luhrs T Ritter C Adrian M Riek-Loher D Bohrmann B Dobeli H 3D structure of Alzheimer's amyloid-beta(1-42) fibrils Proc Natl Acad Sci USA 2005 102 (48 17342 17347 16293696 10.1073/pnas.0506723102 PMC1297669

[bib27] Luheshi LM Tartaglia GG Brorsson AC Pawar AP Watson IE Chiti F Systematic *in vivo* analysis of the intrinsic determinants of amyloid Beta pathogenicity PLoS Biol 2007 5 (11 e290 17973577 10.1371/journal.pbio.0050290 PMC2043051

[bib28] Ward RV Jennings KH Jepras R Neville W Owen DE Hawkins J Fractionation and characterization of oligomeric, protofibrillar and fibrillar forms of beta-amyloid peptide Biochem J 2000 348 (Pt 1 137 144 10794724 PMC1221046

[bib29] Jarrett JT Berger EP Lansbury PT Jr. The carboxy terminus of the beta amyloid protein is critical for the seeding of amyloid formation: implications for the pathogenesis of Alzheimer's disease Biochemistry 1993 32 (18 4693 4697 8490014 10.1021/bi00069a001

[bib30] Sabate R Estelrich J Evidence of the existence of micelles in the fibrillogenesis of beta-amyloid peptide J Phys Chem B 2005 109 (21 11027 11032 16852343 10.1021/jp050716m

[bib31] Lomakin A Teplow DB Kirschner DA Benedek GB Kinetic theory of fibrillogenesis of amyloid beta-protein Proc Natl Acad Sci USA 1997 94 (15 7942 7947 9223292 10.1073/pnas.94.15.7942 PMC21534

[bib32] Lee J Culyba EK Powers ET Kelly JW Amyloid-beta forms fibrils by nucleated conformational conversion of oligomers Nat Chem Biol 2011 7 (9 602 609 21804535 10.1038/nchembio.624 PMC3158298

[bib33] Lambert MP Barlow AK Chromy BA Edwards C Freed R Liosatos M Diffusible, nonfibrillar ligands derived from Abeta1-42 are potent central nervous system neurotoxins Proc Natl Acad Sci USA 1998 95 (11 6448 6453 9600986 10.1073/pnas.95.11.6448 PMC27787

[bib34] McLean CA Cherny RA Fraser FW Fuller SJ Smith MJ Beyreuther K Soluble pool of Abeta amyloid as a determinant of severity of neurodegeneration in Alzheimer's disease Ann Neurol 1999 46 (6 860 866 10589538 10.1002/1531-8249(199912)46:6<860::aid-ana8>3.0.co;2-m

[bib35] Mc Donald JM Savva GM Brayne C Welzel AT Forster G Shankar GM The presence of sodium dodecyl sulphate-stable Abeta dimers is strongly associated with Alzheimer-type dementia Brain 2010 133 (Pt 5 1328 1341 20403962 10.1093/brain/awq065 PMC2859152

[bib36] Williams TL Day IJ Serpell LC The effect of Alzheimer's Abeta aggregation state on the permeation of biomimetic lipid vesicles Langmuir 2010 26 (22 17260 17268 20923185 10.1021/la101581g

[bib37] Narayan P Ganzinger KA McColl J Weimann L Meehan S Qamar S Single molecule characterization of the interactions between amyloid-beta peptides and the membranes of hippocampal cells J Am Chem Soc 2013 135 (4 1491 1498 23339742 10.1021/ja3103567 PMC3561772

[bib38] Chafekar SM Baas F Scheper W Oligomer-specific Abeta toxicity in cell models is mediated by selective uptake Biochim Biophys Acta 2008 1782 (9 523 531 18602001 10.1016/j.bbadis.2008.06.003

[bib39] Bezprozvanny I Amyloid goes global Sci Signal 2009 2 (63 pe16 19318622 10.1126/scisignal.263pe16 PMC4996660

[bib40] Perrin RJ Fagan AM Holtzman DM Multimodal techniques for diagnosis and prognosis of Alzheimer's disease Nature 2009 461 (7266 916 922 19829371 10.1038/nature08538 PMC2810658

[bib41] Selkoe DJ Amyloid protein and Alzheimer's disease Sci Am 1991 265 (5 68 678. 1785042 10.1038/scientificamerican1191-68

[bib42] Williams TL Serpell LC Membrane and surface interactions of Alzheimer's Abeta peptide—insights into the mechanism of cytotoxicity FEBS J 2011 278 (20 3905 3917 21722314 10.1111/j.1742-4658.2011.08228.x

[bib43] Fandrich M Oligomeric intermediates in amyloid formation: structure determination and mechanisms of toxicity J Mol Biol 2012 421 (4-5 427 440 22248587 10.1016/j.jmb.2012.01.006

[bib44] Wang J Dickson DW Trojanowski JQ Lee VM The levels of soluble versus insoluble brain Abeta distinguish Alzheimer's disease from normal and pathologic aging Exp Neurol 1999 158 (2 328 337 10415140 10.1006/exnr.1999.7085

[bib45] Takahashi RH Capetillo-Zarate E Lin MT Milner TA Gouras GK Accumulation of intraneuronal beta-amyloid 42 peptides is associated with early changes in microtubule-associated protein 2 in neurites and synapses PLoS One 2013 8 (1 e51965 23372648 10.1371/journal.pone.0051965 PMC3553177

[bib46] Lacor PN Buniel MC Furlow PW Clemente AS Velasco PT Wood M Abeta oligomer-induced aberrations in synapse composition, shape, and density provide a molecular basis for loss of connectivity in Alzheimer's disease J Neurosci 2007 27 (4 796 807 17251419 10.1523/JNEUROSCI.3501-06.2007 PMC6672917

[bib47] Funato H Enya M Yoshimura M Morishima-Kawashima M Ihara Y Presence of sodium dodecyl sulfate-stable amyloid beta-protein dimers in the hippocampus CA1 not exhibiting neurofibrillary tangle formation Am J Pathol 1999 155 (1 23 28 10393832 10.1016/s0002-9440(10)65094-8 PMC1866667

[bib48] Cleary JP Walsh DM Hofmeister JJ Shankar GM Kuskowski MA Selkoe DJ Natural oligomers of the amyloid-beta protein specifically disrupt cognitive function Nat Neurosci 2005 8 (1 79 84 15608634 10.1038/nn1372

[bib49] Liu RQ Zhou QH Ji SR Zhou Q Feng D Wu Y Membrane localization of beta-amyloid 1-42 in lysosomes: a possible mechanism for lysosome labilization J Biol Chem 2010 285 (26 19986 19996 20430896 10.1074/jbc.M109.036798 PMC2888410

[bib50] Soura V Stewart-Parker M Williams TL Ratnayaka A Atherton J Gorringe K Visualization of co-localization in Abeta42-administered neuroblastoma cells reveals lysosome damage and autophagosome accumulation related to cell death Biochem J 2012 441 (2 579 590 21955321 10.1042/BJ20110749

[bib51] Wu F Yao PJ Clathrin-mediated endocytosis and Alzheimer's disease: an update Ageing Res Rev 2009 8 (3 147 149 19491039 10.1016/j.arr.2009.03.002

[bib52] Song MS Baker GB Todd KG Kar S Inhibition of beta-amyloid1-42 internalization attenuates neuronal death by stabilizing the endosomal-lysosomal system in rat cortical cultured neurons Neuroscience 2011 178 181 188 21262324 10.1016/j.neuroscience.2010.12.055

[bib53] Dutescu RM Li QX Crowston J Masters CL Baird PN Culvenor JG Amyloid precursor protein processing and retinal pathology in mouse models of Alzheimer's disease Graefes Arch Clin Exp Ophthalmol 2009 247 (9 1213 1221 19271231 10.1007/s00417-009-1060-3

[bib54] Johnson LV Leitner WP Rivest AJ Staples MK Radeke MJ Anderson DH The Alzheimer's A beta -peptide is deposited at sites of complement activation in pathologic deposits associated with aging and age-related macular degeneration Proc Natl Acad Sci USA 2002 99 (18 11830 11835 12189211 10.1073/pnas.192203399 PMC129354

[bib55] Prakasam A Muthuswamy A Ablonczy Z Greig NH Fauq A Rao KJ Differential accumulation of secreted AbetaPP metabolites in ocular fluids J Alzheimers Dis 2010 20 (4 1243 1253 20413851 10.3233/JAD-2010-100210 PMC3397687

[bib56] Wang J Ohno-Matsui K Morita I Elevated amyloid beta production in senescent retinal pigment epithelium, a possible mechanism of subretinal deposition of amyloid beta in age-related macular degeneration Biochem Biophys Res Commun 2012 423 (1 73 78 22634014 10.1016/j.bbrc.2012.05.085

[bib57] Anderson DH Talaga KC Rivest AJ Barron E Hageman GS Johnson LV Characterization of beta amyloid assemblies in drusen: the deposits associated with aging and age-related macular degeneration Exp Eye Res 2004 78 (2 243 256 14729357 10.1016/j.exer.2003.10.011

[bib58] Seubert P Vigo-Pelfrey C Esch F Lee M Dovey H Davis D Isolation and quantification of soluble Alzheimer's beta-peptide from biological fluids Nature 1992 359 (6393 325 327 1406936 10.1038/359325a0

[bib59] Moghekar A Rao S Li M Ruben D Mammen A Tang X Large quantities of Abeta peptide are constitutively released during amyloid precursor protein metabolism *in vivo* and *in vitro* J Biol Chem 2011 286 (18 15989 15997 21454701 10.1074/jbc.M110.191262 PMC3091208

[bib60] Hoh KJ Lenassi E Jeffery G Viewing ageing eyes: diverse sites of amyloid Beta accumulation in the ageing mouse retina and the up-regulation of macrophages PLoS One 2010 5 (10 e13127 20957206 10.1371/journal.pone.0013127 PMC2948519

[bib61] Luibl V Isas JM Kayed R Glabe CG Langen R Chen J Drusen deposits associated with aging and age-related macular degeneration contain nonfibrillar amyloid oligomers J Clin Invest 2006 116 (2 378 385 16453022 10.1172/JCI25843 PMC1359048

[bib62] Berisha F Feke GT Trempe CL McMeel JW Schepens CL Retinal abnormalities in early Alzheimer's disease Invest Ophthalmol Vis Sci 2007 48 (5 2285 2289 17460292 10.1167/iovs.06-1029

[bib63] Liu RT Gao J Cao S Sandhu N Cui JZ Chou CL Inflammatory mediators induced by amyloid-beta in the retina and RPE *in vivo*: implications for inflammasome activation in age-related macular degeneration Invest Ophthalmol Vis Sci 2013 54 (3 2225 2237 23462752 10.1167/iovs.12-10849 PMC3947398

[bib64] Isas JM Luibl V Johnson LV Kayed R Wetzel R Glabe CG Soluble and mature amyloid fibrils in drusen deposits Invest Ophthalmol Vis Sci 2010 51 (3 1304 1310 19892876 10.1167/iovs.09-4207 PMC2840723

[bib65] Dentchev T Milam AH Lee VM Trojanowski JQ Dunaief JL Amyloid-beta is found in drusen from some age-related macular degeneration retinas, but not in drusen from normal retinas Mol Vis 2003 9 184 190 12764254

[bib66] Kurji KH Cui JZ Lin T Harriman D Prasad SS Kojic L Microarray analysis identifies changes in inflammatory gene expression in response to amyloid-beta stimulation of cultured human retinal pigment epithelial cells Invest Ophthalmol Vis Sci 2010 51 (2 1151 1163 19797223 10.1167/iovs.09-3622 PMC3947389

[bib67] Cao L Wang H Wang F Xu D Liu F Liu C Abeta-induced senescent retinal pigment epithelial cells create a proinflammatory microenvironment in AMD Invest Ophthalmol Vis Sci 2013 54 (5 3738 3750 23557734 10.1167/iovs.13-11612

[bib68] Holtkamp GM Kijlstra A Peek R de Vos AF Retinal pigment epithelium-immune system interactions: cytokine production and cytokine-induced changes Prog Retin Eye Res 2001 20 (1 29 48 11070367 10.1016/s1350-9462(00)00017-3

[bib69] Yang D Elner SG Bian ZM Till GO Petty HR Elner VM Pro-inflammatory cytokines increase reactive oxygen species through mitochondria and NADPH oxidase in cultured RPE cells Exp Eye Res 2007 85 (4 462 472 17765224 10.1016/j.exer.2007.06.013 PMC2094037

[bib70] Liu XC Liu XF Jian CX Li CJ He SZ IL-33 is induced by amyloid-beta stimulation and regulates inflammatory cytokine production in retinal pigment epithelium cells Inflammation 2012 35 (2 776 784 21898270 10.1007/s10753-011-9379-4

[bib71] Bruban J Glotin AL Dinet V Chalour N Sennlaub F Jonet L Amyloid-beta(1-42) alters structure and function of retinal pigmented epithelial cells Aging Cell 2009 8 (2 162 177 19239420 10.1111/j.1474-9726.2009.00456.x

[bib72] Tarallo V Hirano Y Gelfand BD Dridi S Kerur N Kim Y DICER1 loss and Alu RNA induce age-related macular degeneration via the NLRP3 inflammasome and MyD88 Cell 2012 149 (4 847 859 22541070 10.1016/j.cell.2012.03.036 PMC3351582

[bib73] Tsuruma K Tanaka Y Shimazawa M Hara H Induction of amyloid precursor protein by the neurotoxic peptide, amyloid-beta 25-35, causes retinal ganglion cell death J Neurochem 2010 113 (6 1545 1554 20374419 10.1111/j.1471-4159.2010.06724.x

[bib74] Guo L Salt TE Luong V Wood N Cheung W Maass A Targeting amyloid-beta in glaucoma treatment Proc Natl Acad Sci USA 2007 104 (33 13444 13449 17684098 10.1073/pnas.0703707104 PMC1940230

[bib75] Khandhadia S Cipriani V Yates JR Lotery AJ Age-related macular degeneration and the complement system Immunobiology 2012 217 (2 127 146 21868123 10.1016/j.imbio.2011.07.019

[bib76] Anderson DH Radeke MJ Gallo NB Chapin EA Johnson PT Curletti CR The pivotal role of the complement system in aging and age-related macular degeneration: hypothesis re-visited Prog Retin Eye Res 2010 29 (2 95 112 19961953 10.1016/j.preteyeres.2009.11.003 PMC3641842

[bib77] Bradt BM Kolb WP Cooper NR Complement-dependent proinflammatory properties of the Alzheimer's disease beta-peptide J Exp Med 1998 188 (3 431 438 9687521 10.1084/jem.188.3.431 PMC2212467

[bib78] Akiyama H Barger S Barnum S Bradt B Bauer J Cole GM Inflammation and Alzheimer's disease Neurobiol Aging 2000 21 (3 383 421 10858586 10.1016/s0197-4580(00)00124-x PMC3887148

[bib79] Hageman GS Anderson DH Johnson LV Hancox LS Taiber AJ Hardisty LI A common haplotype in the complement regulatory gene factor H (HF1/CFH) predisposes individuals to age-related macular degeneration Proc Natl Acad Sci USA 2005 102 (20 7227 7232 15870199 10.1073/pnas.0501536102 PMC1088171

[bib80] Parks WC Wilson CL Lopez-Boado YS Matrix metalloproteinases as modulators of inflammation and innate immunity Nat Rev Immunol 2004 4 (8 617 629 15286728 10.1038/nri1418

[bib81] Butterfield DA Boyd-Kimball D Amyloid beta-peptide(1-42) contributes to the oxidative stress and neurodegeneration found in Alzheimer disease brain Brain Pathol 2004 14 (4 426 432 15605990 10.1111/j.1750-3639.2004.tb00087.x PMC8096022

[bib82] Kinnunen K Petrovski G Moe MC Berta A Kaarniranta K Molecular mechanisms of retinal pigment epithelium damage and development of age-related macular degeneration Acta Ophthalmol 2012 90 (4 299 309 22112056 10.1111/j.1755-3768.2011.02179.x

[bib83] Blasiak J Glowacki S Kauppinen A Kaarniranta K Mitochondrial and nuclear DNA damage and repair in age-related macular degeneration Int J Mol Sci 2013 14 (2 2996 3010 23434654 10.3390/ijms14022996 PMC3588027

[bib84] Cunvong K Huffmire D Ethell DW Cameron DJ Amyloid-beta increases capillary bed density in the adult zebrafish retina Invest Ophthalmol Vis Sci 2013 54 (2 1516 1521 23404118 10.1167/iovs.12-10821

[bib85] Fritsche LG Fariss RN Stambolian D Abecasis GR Curcio CA Swaroop A Age-related macular degeneration: genetics and biology coming together Annu Rev Genomics Hum Genet 2014 15 151 171 24773320 10.1146/annurev-genom-090413-025610 PMC4217162

[bib86] Biron KE Dickstein DL Gopaul R Jefferies WA Amyloid triggers extensive cerebral angiogenesis causing blood brain barrier permeability and hypervascularity in Alzheimer's disease PLoS One 2011 6 (8 e23789 21909359 10.1371/journal.pone.0023789 PMC3166122

[bib87] McKay GJ Patterson CC Chakravarthy U Dasari S Klaver CC Vingerling JR Evidence of association of APOE with age-related macular degeneration: a pooled analysis of 15 studies Hum Mutat 2011 32 (12 1407 1416 21882290 10.1002/humu.21577 PMC3217135

[bib88] Curcio CA Johnson M Rudolf M Huang JD The oil spill in ageing Bruch membrane Br J Ophthalmol 2011 95 (12 1638 1645 21890786 10.1136/bjophthalmol-2011-300344 PMC3633599

[bib89] Loane E McKay GJ Nolan JM Beatty S Apolipoprotein E genotype is associated with macular pigment optical density Invest Ophthalmol Vis Sci 2010 51 (5 2636 2643 20107178 10.1167/iovs.09-4397

[bib90] Strittmatter WJ Weisgraber KH Huang DY Dong LM Salvesen GS Pericak-Vance M Binding of human apolipoprotein E to synthetic amyloid beta peptide: isoform-specific effects and implications for late-onset Alzheimer disease Proc Natl Acad Sci USA 1993 90 (17 8098 8102 8367470 10.1073/pnas.90.17.8098 PMC47295

[bib91] Castellano JM Kim J Stewart FR Jiang H DeMattos RB Patterson BW Human apoE isoforms differentially regulate brain amyloid-beta peptide clearance Sci Transl Med 2011 3 (89 89ra57 10.1126/scitranslmed.3002156 PMC3192364 21715678

[bib92] Cerf E Gustot A Goormaghtigh E Ruysschaert JM Raussens V High ability of apolipoprotein E4 to stabilize amyloid-beta peptide oligomers, the pathological entities responsible for Alzheimer's disease FASEB J 2011 25 (5 1585 1595 21266538 10.1096/fj.10-175976

[bib93] Ricciarelli R Canepa E Marengo B Marinari UM Poli G Pronzato MA Cholesterol and Alzheimer's disease: a still poorly understood correlation IUBMB Life 2012 64 (12 931 935 23124820 10.1002/iub.1091

[bib94] Castello MA Soriano S Rational heterodoxy: cholesterol reformation of the amyloid doctrine Ageing Res Rev 2013 12 (1 282 288 22771381 10.1016/j.arr.2012.06.007

[bib95] Wang J Ohno-Matsui K Morita I Cholesterol enhances amyloid beta deposition in mouse retina by modulating the activities of Abeta-regulating enzymes in retinal pigment epithelial cells Biochem Biophys Res Commun 2012 424 (4 704 709 22796523 10.1016/j.bbrc.2012.07.014

[bib96] Dasari B Prasanthi JR Marwarha G Singh BB Ghribi O Cholesterol-enriched diet causes age-related macular degeneration-like pathology in rabbit retina BMC Ophthalmol 2011 11 22 21851605 10.1186/1471-2415-11-22 PMC3170645

[bib97] Malek G Johnson LV Mace BE Saloupis P Schmechel DE Rickman DW Apolipoprotein E allele-dependent pathogenesis: a model for age-related retinal degeneration Proc Natl Acad Sci USA 2005 102 (33 11900 11905 16079201 10.1073/pnas.0503015102 PMC1187976

[bib98] Ding JD Johnson LV Herrmann R Farsiu S Smith SG Groelle M Anti-amyloid therapy protects against retinal pigmented epithelium damage and vision loss in a model of age-related macular degeneration Proc Natl Acad Sci USA 2011 108 (28 E279 E287 21690377 10.1073/pnas.1100901108 PMC3136266

[bib99] Parisi V Restuccia R Fattapposta F Mina C Bucci MG Pierelli F Morphological and functional retinal impairment in Alzheimer's disease patients Clin Neurophysiol 2001 112 (10 1860 1867 11595144 10.1016/s1388-2457(01)00620-4

[bib100] Gharbiya M Trebbastoni A Parisi F Manganiello S Cruciani F D'Antonio F Choroidal thinning as a new finding in Alzheimer's disease: evidence from enhanced depth imaging spectral domain optical coherence tomography J Alzheimers Dis 2014 40 (4 907 917 24577467 10.3233/JAD-132039

[bib101] Morris KL Rodger A Hicks MR Debulpaep M Schymkowitz J Rousseau F Exploring the sequence-structure relationship for amyloid peptides Biochem J 2013 450 275 283 23252554 10.1042/BJ20121773 PMC3573774

